# Umbilical Hernia Probe-Induced Cocco Sign: Color Doppler During Pressure and Release in Standing Position

**DOI:** 10.3390/diagnostics15222863

**Published:** 2025-11-12

**Authors:** Corrado Tagliati, Marco Fogante, Claudio Ventura, Stefania Lamja, Roberto Esposito, Marco Di Serafino, Antonio Corvino, Giulio Argalia, Ernesto Di Cesare, Andrea Delli Pizzi, Giulio Cocco

**Affiliations:** 1 AST Ancona, Ospedale di Comunità Maria Montessori di Chiaravalle, Via Fratelli Rosselli 176, 60033 Chiaravalle, Italy; 2 Maternal-Child, Senological, Cardiological Radiology and Outpatient Ultrasound, Department of Radiological Sciences, University Hospital of Marche, Via Conca 71, 60126 Ancona, Italy; claudioventura20@gmail.com (C.V.); giulio.argalia@gmail.com (G.A.); 3 Department of Biotechnological and Applied Clinical Sciences, University of L’Aquila, Via Vetoio, 67100 L’Aquila, Italy; stefanialamja@gmail.com (S.L.); ernesto.dicesare@univaq.it (E.D.C.); 4 Gemini Med Diagnostic Clinic, Strada Rovereta 42, 47891 Falciano, San Marino; resposito1979@gmail.com; 5 Department of Radiology, Cardarelli Hospital, 80131 Naples, Italy; marcodiserafino@hotmail.it; 6 Medical, Movement and Wellbeing Sciences Department, University of Naples “Parthenope”, 80133 Naples, Italy; an.cor@hotmail.it; 7 Department of Innovative Technologies in Medicine and Dentistry, University “G. d’Annunzio”, 66100 Chieti, Italy; andreadellipizzi@gmail.com; 8 Department of Neuroscience, Imaging and Clinical Sciences, University “G. d’Annunzio”, 66100 Chieti, Italy; cocco.giulio@gmail.com

**Keywords:** Cocco sign, abdominal wall, umbilicus, umbilical hernia, Doppler ultrasound

## Abstract

We describe a case of an ultrasound-detected umbilical hernia in a 56-year-old female patient who underwent a skin ultrasound. With the patient in a standing position, the probe was positioned at the level of the umbilicus. Pressure was applied with the probe and then it was released; probe-induced Cocco sign was revealed.

**Figure 1:**
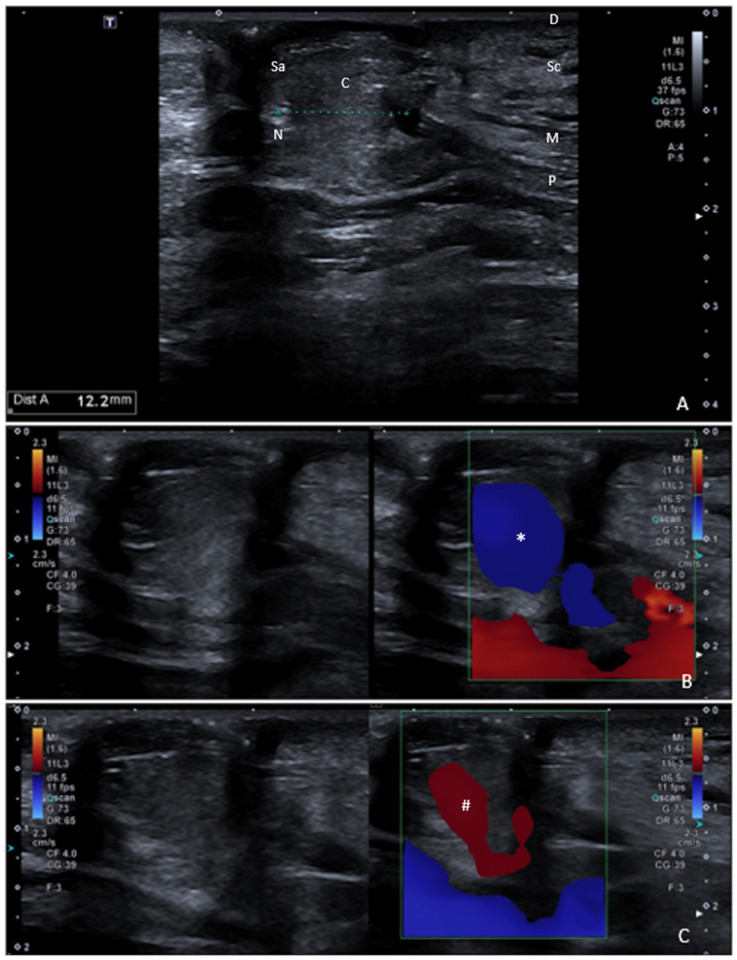
Linear probe B-mode ultrasound imaging showed umbilical hernia (**A**), with three key elements: sac (Sa), neck (N), and content (C) [1]. In our patient, the content consisted of omental fat; however, hernias can contain other tissues too, such as small and/or large bowel loops [2]. Anterior abdominal wall superficial layer consists of very thin epidermis, thicker dermis (D), and underlying subcutis (Su). Myofascial layer is the middle one, and consists of muscles and their fascial envelopes (M). Transversalis fascia, preperitoneal fat, and the parietal peritoneum forms the deep layer (P) [3,4,5]. Probe pressure was applied and the hernia partially retracted into the abdominal cavity, generating a blue color on Doppler ultrasound (*, (**B**)). Probe pressure was released, and a red Doppler signal was visible over the hernia, which was moving towards the probe (#, (**C**)). This sequence demonstrated the movement of the hernia, indicating at least a partially reducible hernia under probe pressure (Video S1, Figure S1). In color Doppler mode, when the object is moving away from the transmitter it encounters less oscillations per unit time than its stationary equivalent, so the frequency of the reflected wave is apparently reduced; instead, when the object is moving towards the ultrasound wave, the frequency of the reflected wave is increased. Usually, the reduction and increase in the reflected waves frequency are, respectively, depicted with blue and red colors in the image [6,7,8,9]. As the patient stands up still, the probe is gently placed over the hernia site without compression and the Dual Image (B-mode/color Doppler) is activated, setting the Pulse Repetition Frequency to about 15 cm/s. During the probe pressure, the hernia sac motion through the hernial gap appears as a blue color Doppler signal moving away from the probe, indicating at least a partially reducible hernia. When the probe pressure is released, the hernia sac motion toward the probe causes the appearance of a red Doppler signal over the hernia. Instead, as the patient lies in a supine position, the Cocco sign consists of the detection of the movement toward the probe of the abdominal wall hernia sac during the Valsalva maneuver, and of its retraction upon the cessation of the Valsalva maneuver [10]. Therefore, there are some differences between probe-induced Cocco sign and the Cocco sign. First, in the former, the patient stands up, whereas in the Cocco sign the patient lies in a supine position. Second, in the former, the patient does not need to do anything, whereas the Valsalva maneuver is required in the Cocco sign. Third, in the former, the pressure applied to the probe determines the movement of the hernia if it is at least partially reducible. Fourth, the cessation of the probe pressure allows the hernia to move forward again. It should be noted that the Valsalva maneuver can be related to some limitations in the Cocco sign technique, such as in patients who are unable to perform the Valsalva maneuver due to respiratory or other medical conditions. Therefore, the probe-induced Cocco sign can overcome these limitations, and, to the best of our knowledge, this is the first report about probe-induced Cocco sign.

## Data Availability

The original contributions presented in this study are included in the article/Supplementary Material. Further inquiries can be directed to the corresponding authors.

## Supplementary Materials

The following supporting information can be downloaded at: https://www.mdpi.com/article/10.3390/diagnostics15222863/s1. Video S1: Umbilical hernia probe-induced Cocco sign video showing blue and red color Doppler signals over the hernia during pressure and release in standing position. Figure S1: Umbilical hernia probe-induced Cocco sign with blue and red color Doppler signals over the hernia during pressure and release in standing position using another older ultrasound scanner.
