# Supplementary figures and images for: Umbilical Hernia Probe-Induced Cocco Sign: Color Doppler During Pressure and Release in Standing Position
Source: Diagnostics (Basel). 2025 Nov 12;15(22):2863. doi: 10.3390/diagnostics15222863 (PMC12650979; doi:10.3390/diagnostics15222863)

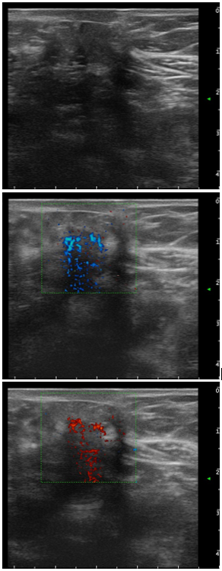

Supplement: Supplementary file 1 [file diagnostics-15-02863-s001.zip › Figure S1.png]
